# Feedstock-Informed Technology Selection for Chemical Recycling of Plastic Rejects: An Integrated Physicochemical Characterization and Multi-Criteria Decision Framework

**DOI:** 10.3390/polym18212628

**Published:** 2026-10-23

**Authors:** Joseph F. Mtabazi, Jacob M. Kihila, Minza I. Selele, Liberatha Kawamala

**Affiliations:** 1 Department of Civil and Environmental Engineering, Ardhi University, Dar es Salaam P.O. Box 35176, Tanzania; minza.selele@gmail.com; 2 Institute of Human Settlements Studies, Ardhi University, Dar es Salaam P.O. Box 35176, Tanzania; jacob.kihila@aru.ac.tz; 3 Libe Green Innovation, Dar es Salaam P.O. Box 13027, Tanzania; liberathakawamala@gmail.com

**Keywords:** plastic rejects, chemical recycling, Material Recovery Facility, gasification, AHP-TOPSIS, circular economy

## Abstract

Rapid urbanization in the Global South has intensified plastic waste management challenges, and limited empirical data on plastic waste characteristics continue to hinder the adoption of appropriate recovery technologies in Africa. This study characterized non-recyclable plastic rejects from the Libegreen Innovation Material Recovery Facility (MRF) in Dar es Salaam, Tanzania, and evaluated suitable chemical recycling pathways. An integrated analytical approach combining material flow analysis, Fourier-transform infrared spectroscopy (FTIR), proximate and ultimate analysis, calorific value determination, X-ray fluorescence (XRF), X-ray diffraction (XRD), and thermogravimetric analysis (TGA) was used to determine the physicochemical and thermal properties. A hybrid Analytic Hierarchy Process and Technique for Order of Preference by Similarity to Ideal Solution (AHP-TOPSIS) multi-criteria decision framework was used to compare pyrolysis, gasification, and depolymerization technologies. The results showed that the Material Recovery Facility achieved a high material recovery efficiency of 95.5%, with only 4.5% (181 tons/year) rejected, resulted in an estimated annual economic loss of USD 34,774–62,632. The rejects were dominated by polypropylene (51.58%), polyethylene terephthalate (28.42%), and polyvinyl chloride (20%). Polypropylene exhibited the highest energy recovery potential (28.82 MJ/kg HHV), whereas polyvinyl chloride presented processing challenges due to high chlorine content (36.7 wt%) and dehydrochlorination at approximately 280 °C. AHP-TOPSIS ranked gasification as the most suitable pathway (Ri = 0.718), followed by pyrolysis (Ri = 0.517) and then depolymerization (Ri = 0.196). These findings provide empirical evidence for advancing chemical recycling strategies and transforming plastic rejects into valuable resources within emerging circular economies.

## 1. Introduction

The rapid growth in global plastic production and consumption has created a persistent waste management challenge, particularly within the rapidly urbanizing regions of the Global South [1,2]. In addition to waste accumulation, inadequately managed plastics block drainage systems, leading to flooding, land and water pollution, and threatening human health [3,4]. In sub-Saharan Africa, limited source separation and inadequate collection systems have resulted in large volumes of plastic waste being disposed of in open dumps or aquatic environments, exacerbating flooding and human health risks [5,6,7]. This issue is particularly severe for mixed and low-value plastic types, which have minimal recycling potential but persist in the environment.

In response to these challenges, various policy and technological interventions have been implemented in the region, including single-use plastic restrictions, municipal waste collection campaigns, and the establishment of manual or semi-automated MRFs. However, these interventions remain constrained by inadequate enforcement, limited collection infrastructure, insufficient sorting capacity, and financial constraints. Moreover, Material Recovery Facilities (MRFs) serve as a critical intervention by diverting plastics from final disposal for mechanical recycling [8]. However, even well-operated MRFs generate substantial quantities of plastic rejects, multilayer films, contaminated packaging, and low-grade polymers that do not meet the quality requirements for mechanical recycling [9]. Thus, although MRFs reduce the recyclable fraction, they leave a residual stream that is difficult to manage. In cities such as Dar es Salaam, these residual fractions represent an emerging waste management concern as they are directed to landfills or subjected to uncontrolled burning. Landfilling does not recover the material or energy value of plastics, whereas uncontrolled burning can generate harmful emissions, demonstrating the need for alternative treatment pathways [10,11]. This reduces the overall effectiveness of recycling initiatives and contributes to environmental pollution.

Mechanical recycling remains the predominant method for plastic resource recovery. However, its implementation in numerous countries has encountered substantial technical and economic challenges [12]. The success of mechanical recycling is largely contingent on the availability of clean, well-sorted single-polymer waste streams. Furthermore, repeated mechanical processing induces polymer degradation, which diminishes the material quality and restricts the number of recycling cycles [13,14,15]. Recyclability is further compromised by the presence of multilayer packaging, additives, pigments, and mixed polymer compositions, which also curtail the market demand for recovered materials [16]. These limitations are especially relevant to MRF rejects because their heterogeneous composition, contamination, and low polymer purity make further mechanical processing technically and economically challenging.

Consequently, chemical recycling methods, such as pyrolysis and gasification, offer a transformative pathway for these non-recyclable streams by converting them into fuels or monomers [17,18,19,20]. Unlike mechanical methods, chemical recycling is less constrained by polymer mixing and contamination [21]. Therefore, pyrolysis and gasification can provide routes for recovering the chemical and energy values of plastic rejects while reducing dependence on landfilling and uncontrolled combustion [22,23]. However, their performance depends strongly on the feedstock composition and physicochemical properties [24,25]. Mixed polymers and contaminants can affect product yield, gas composition, energy recovery, and contaminant formation.

Despite this potential, a critical shortage of empirical data on African plastic waste characteristics persists, as most studies rely on generic data from high-income nations [26]. Variations in polymer composition, contamination levels, sorting methods, and material origins suggest that such generalized data fail to accurately reflect the realities of African material recovery facility (MRF) rejects. Consequently, feedstock-specific characterization is essential for selecting appropriate technologies and precisely predicting conversion outcomes. We hypothesize that a comprehensive physicochemical characterization of MRF plastic rejects provides sufficient evidence to systematically identify the optimal chemical recycling pathway using a feedstock-informed multi-criteria decision framework.

## 2. Materials and Methods

### 2.1. Study Area Description

**Figure 1:**
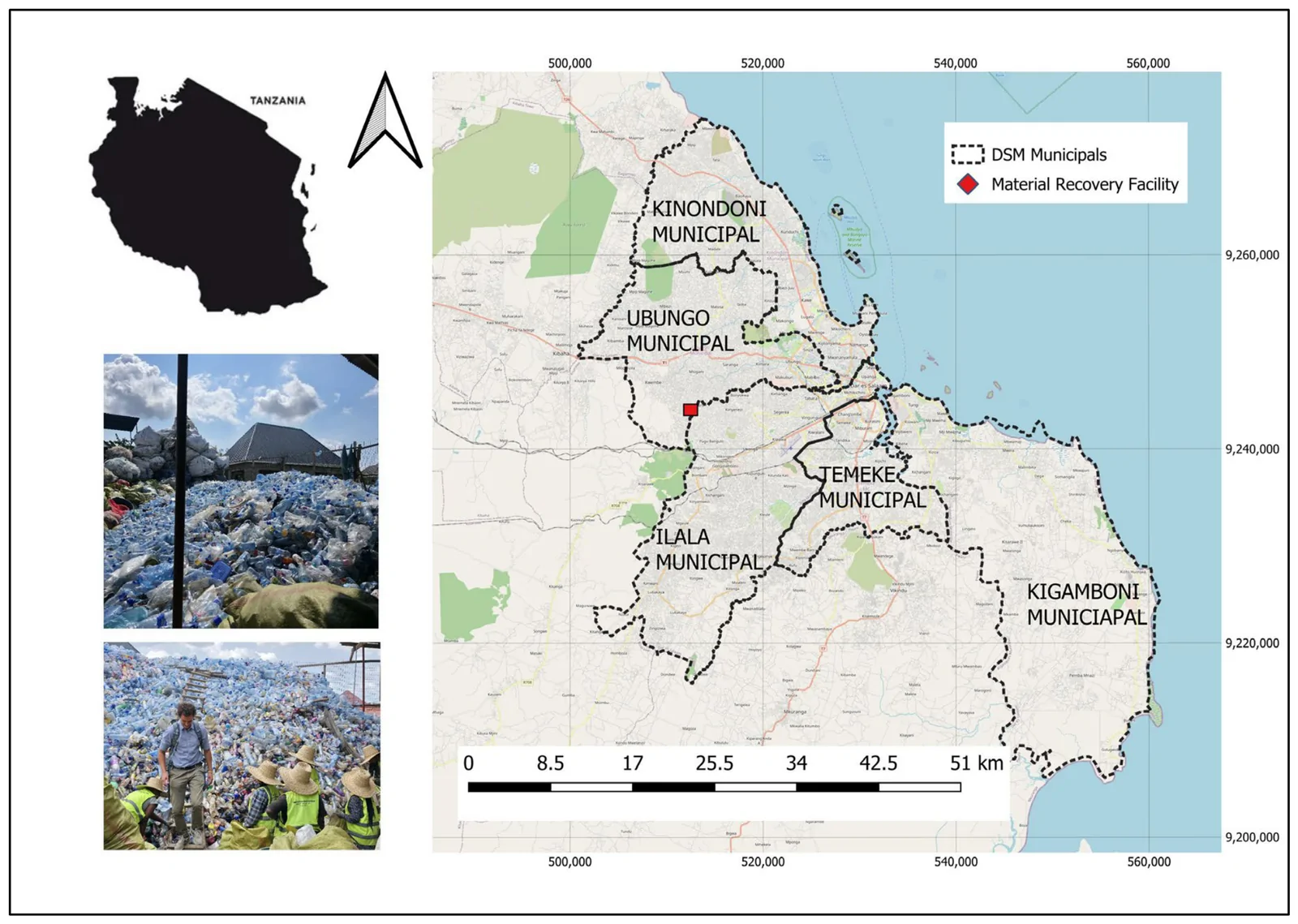
Plastic-Focused Material Recovery Facility (MRF) in Dar es Salaam, Tanzania.

The research was conducted at Libegreen Innovation, a plastic-focused Material Recovery Facility (MRF) situated on Kifuru Street in Kinyerezi Ward, Ubungo Municipality, Dar es Salaam, Tanzania (6.84373° S, 39.12612° E) (Figure 1). Established in 2018, the facility aims to provide sustainable solutions for plastic pollution by recovering and recycling post-consumer plastics, primarily polyethylene terephthalate (PET), high-density polyethylene (HDPE), and polypropylene (PP) plastics. The facility’s infrastructure includes specialized units for waste reception, manual sorting, shredding, and storage, which facilitate the tracking of plastic flows from the initial intake to the final waste. Libegreen Innovation receives its feedstock from a combination of municipal collection systems, waste pickers, and private aggregators, reflecting the broader waste recovery ecosystem in Tanzanian urban centers. This MRF was selected because of its fully operational status and comprehensive historical records of plastic quantities categorized by type and time, providing a robust dataset for material flow analysis, mass balance evaluations, and assessment of recyclable and non-recyclable fractions.

### 2.2. Sample Collection and Preparation

**Figure 2:**
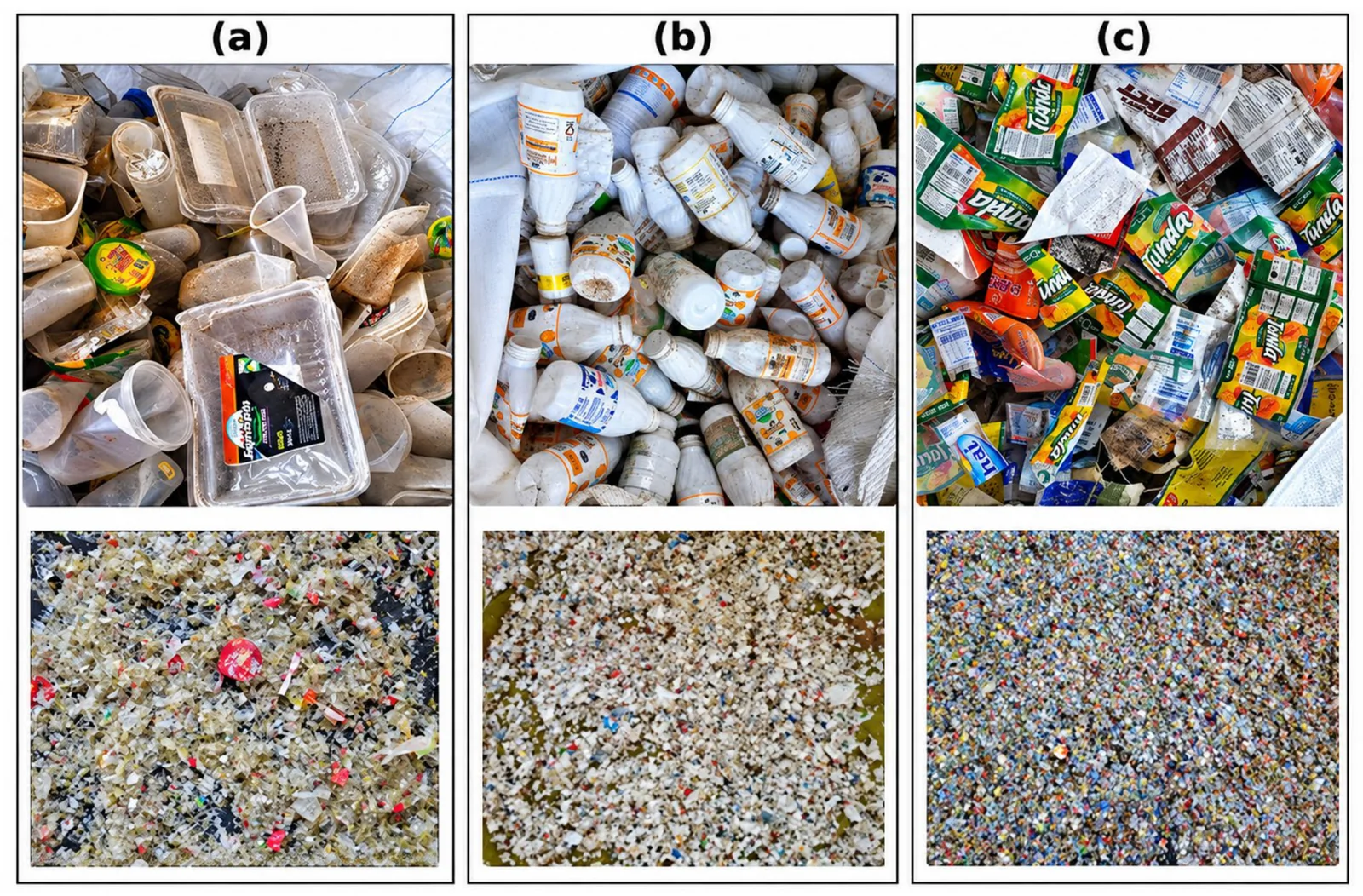
Plastic reject fractions collected from the material recovery facility. (**a**) PP-rich single-use plastic plates and cups before and after size reduction. (**b**) PET-rich milk and yoghurt drink containers before and after size reduction. (**c**) PVC-rich sleeve labels from plastic bottles before and after size reduction.

Sample collection and preparation were conducted over an eight-week period from August to September 2025 at the Libegreen Innovation MRF, with the collected materials stored for seven days prior to their characterization. The process involved the manual segregation of residual plastic waste from sorted remnants through visual inspection of the resin identification code and classification into polymer types, including PET, PVC, and PP, which were subsequently weighed on a calibrated electronic scale (Figure 2). The collected samples were initially shredded into 16 mm pieces at the material recovery facility. To eliminate water-insoluble contaminants, including grease, oils, and fatty residues, the shredded materials were thoroughly washed in a bath containing a 2% *v*/*v* mild non-ionic surfactant solution, followed by a thorough rinse with cold water to remove detergent traces. The clean materials were then air-dried to remove residual moisture. To facilitate detailed physical and chemical characterization, the dried materials were processed into a powdered form using a multifunctional high-speed disintegrator for approximately 2–4 min and subsequently stored in a plastic zipper bag. The shredded samples were packed and transported to Universal Lab in Villigen, Switzerland for physicochemical analysis.

### 2.3. Laboratory and Analytical Methods

An integrated analytical approach was applied to evaluate the physicochemical and thermal characteristics of plastic rejects and their suitability for chemical recycling. Samples of polypropylene (PP), polyethylene terephthalate (PET), polyvinyl chloride (PVC), and mixed plastic rejects were analyzed using complementary techniques. Fourier Transform Infrared Spectroscopy (FTIR) was conducted in Attenuated Total Reflection (ATR) mode using a Shimadzu IRSpirit-T spectrophotometer equipped with a diamond crystal prism. Analyses were performed over a spectral range of 400–4000 cm^−1^ with a resolution of 4 cm^−1^ and an accumulation of 32 scans per sample. Prior to analysis, the instrument underwent automated self-internal checks, and calibration was validated using a polystyrene standard. The PP, PET, PVC, and mixed plastic reject samples were placed onto the clean ATR crystal, ensuring tight contact to achieve optimal infrared wave penetration. A background spectrum of the clean, exposed diamond crystal was recorded after every sample scan to eliminate environmental and system interference. The thermochemical properties were assessed through proximate analysis, ultimate analysis, and calorific value determination. Moisture content, volatile matter, ash content, and fixed carbon were measured to evaluate the conversion behavior, while the carbon, hydrogen, nitrogen, and sulfur contents were determined to assess the fuel quality and emission potential. The higher heating value (HHV) and lower heating value (LHV) were quantified using bomb calorimetry. The Inorganic composition and structural properties were examined using an ARL 9800 Oasis X-ray system (Thermo ARL, Ecublens, Switzerland) combining X-ray fluorescence (XRF) and X-ray diffraction (XRD). XRF was used to identify inorganic fillers, pigments, and contaminants, whereas XRD was used to evaluate polymer crystallinity.

The thermal degradation behavior was investigated using thermogravimetric and differential thermal analyses (TGA/DTA) on a Linseis STA PT 1000 simultaneous thermal analyzer (Linseis Messgeräte GmbH, Selb, Germany) to determine the thermal stability and decomposition profiles to determine the stability and decomposition profiles. To systematically prioritize the evaluation criteria and select the optimal chemical recycling technology for the MRF’s reject stream, a hybrid AHP-TOPSIS multi-criteria decision-making framework was established. This framework integrates the subjective expertise of a purposive panel with objective physical and chemical characterization data of the samples. The framework was executed in the sequence of Analytic Hierarchy Process (AHP) for criteria weighting and Technique for Order Preference by Similarity to Ideal Solution (TOPSIS) for technology alternative ranking. Data analysis and visualization were conducted using Microsoft Excel 2021 (Microsoft Corporation, Redmond, WA, USA) and OriginPro version 8.5 (OriginLab Corporation, Northampton, MA, USA).

## 3. Results

### 3.1. Material Flow and Composition Analysis

#### 3.1.1. Plastic Inflow Composition in a Material Recovery Facility

**Figure 3:**
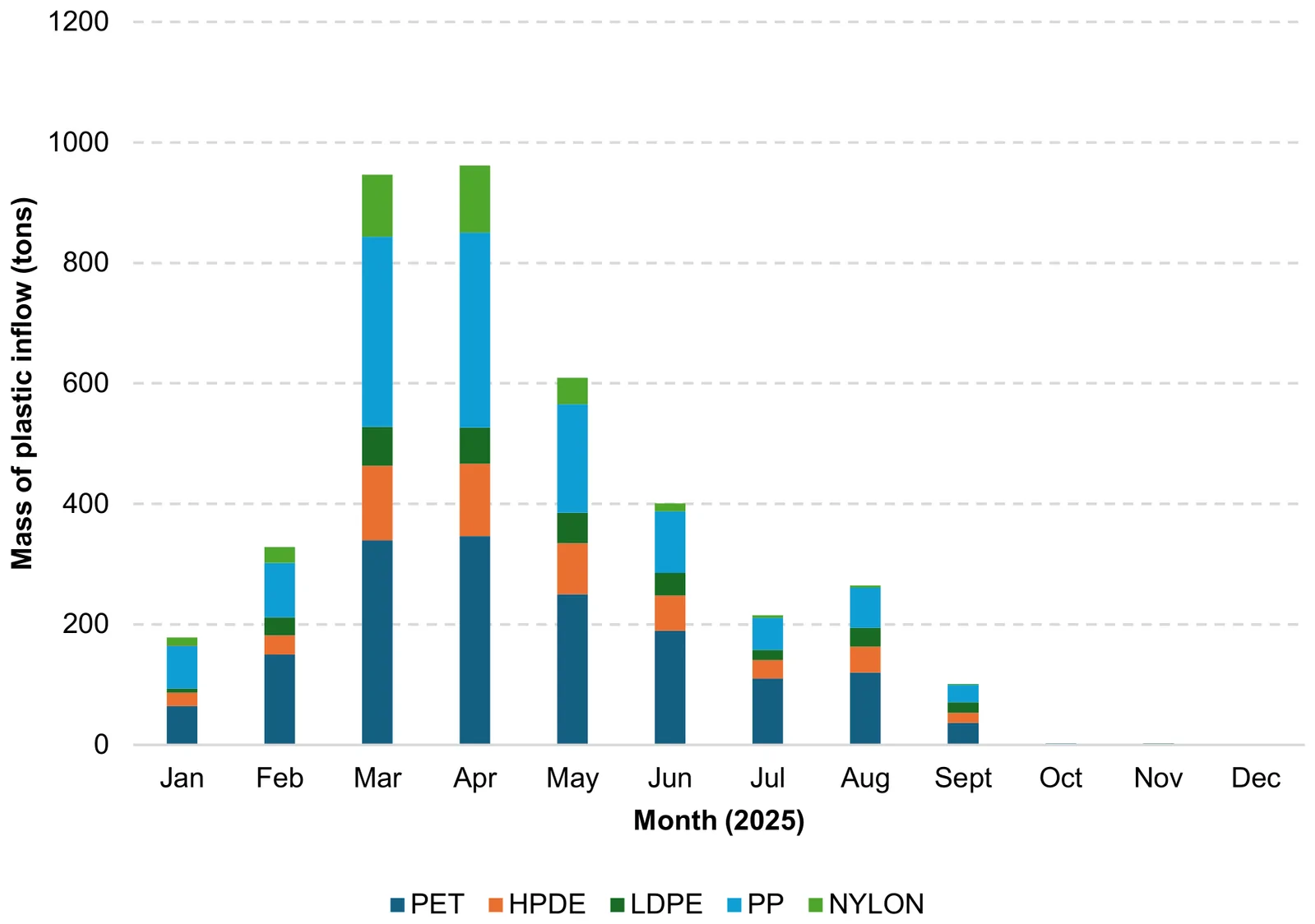
Plastic Inflow at Material Recovery Facility in Dar es Salaam.

**Figure 4:**
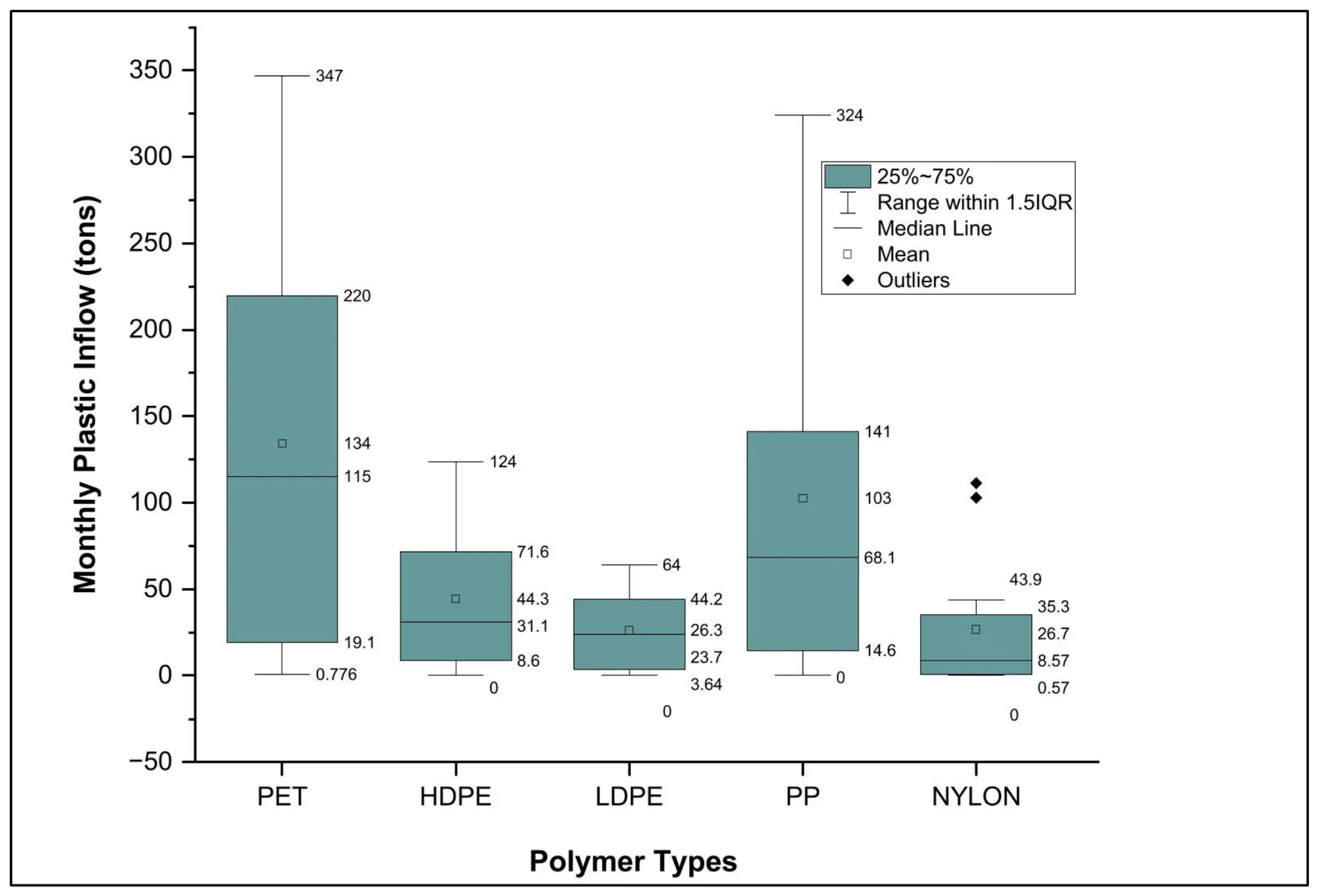
Statistical distribution of monthly plastic inflows at Libegreen Innovation Material Recovery Facility.

The Libegreen Innovation Material Recovery Facility (MRF) received 4010 tons of plastic waste in 2025, with inflow equally supplied by waste pickers/aggregators (32.5%), the Naboli Center (34.7%), and Bwashee Environmental (32.8%). Polymer-specific analysis showed that polyethylene terephthalate (PET) and polypropylene (PP) dominated overall plastic streams, contributing approximately 40% and 31% of the total inflow, respectively, and together accounting for more than 70% of the materials processed. PET recorded the highest annual inflow (1610.51 tons), with an average delivery of 134.21 ± 125.19 tons, followed by PP, with 1231.65 tons annually and an average inflow of 102.64 ± 113.71 tons (Figure 3). Other fractions contributed lower quantities: HDPE (531.52 tons/year; 44.29 ± 44.07 tons/month), nylon (320.46 tons/year; 26.71 ± 39.83 tons/month), and LDPE (315.01 tons/year; 26.25 ± 23.36 tons/month). Moreover, the temporal distribution of plastic inflow demonstrated substantial monthly variability across all polymer categories. Box-and-whisker analysis (Figure 4) revealed positively skewed distributions for the major polymer fractions, particularly PET and PP, where the mean values exceeded their corresponding median values. PET recorded a median monthly inflow of 115.18 tons, compared with a mean of 134.21 tons/month, whereas PP exhibited a median value of 68.14 tons compared with a mean of 102.64 tons/month. The extended upper whiskers observed for PET and PP further confirmed episodic increases in material supply, with maximum monthly inflows reaching 346.89 and 324.02 tons, respectively. In contrast, the lower whiskers reflect periods of limited material availability, particularly toward the end of the year, when several polymer streams decline to near-zero levels.

Furthermore, temporal analysis revealed monthly fluctuations, with peak inflows in March and April. Approximately 62.7% of the plastic supply was received between March and May, indicating intensified recovery despite the rainy season in the city. Deliveries declined between October and December, likely due to disruptions in collection following the election period and the sociopolitical instability. One-way analysis of variance (ANOVA) revealed significant differences among polymer categories (F = 5.86, *p* < 0.001), confirming the dominance of PET and PP in the MRF feedstock.

#### 3.1.2. Plastic Reject Generation and Composition at the Material Recovery Facility

**Figure 5:**
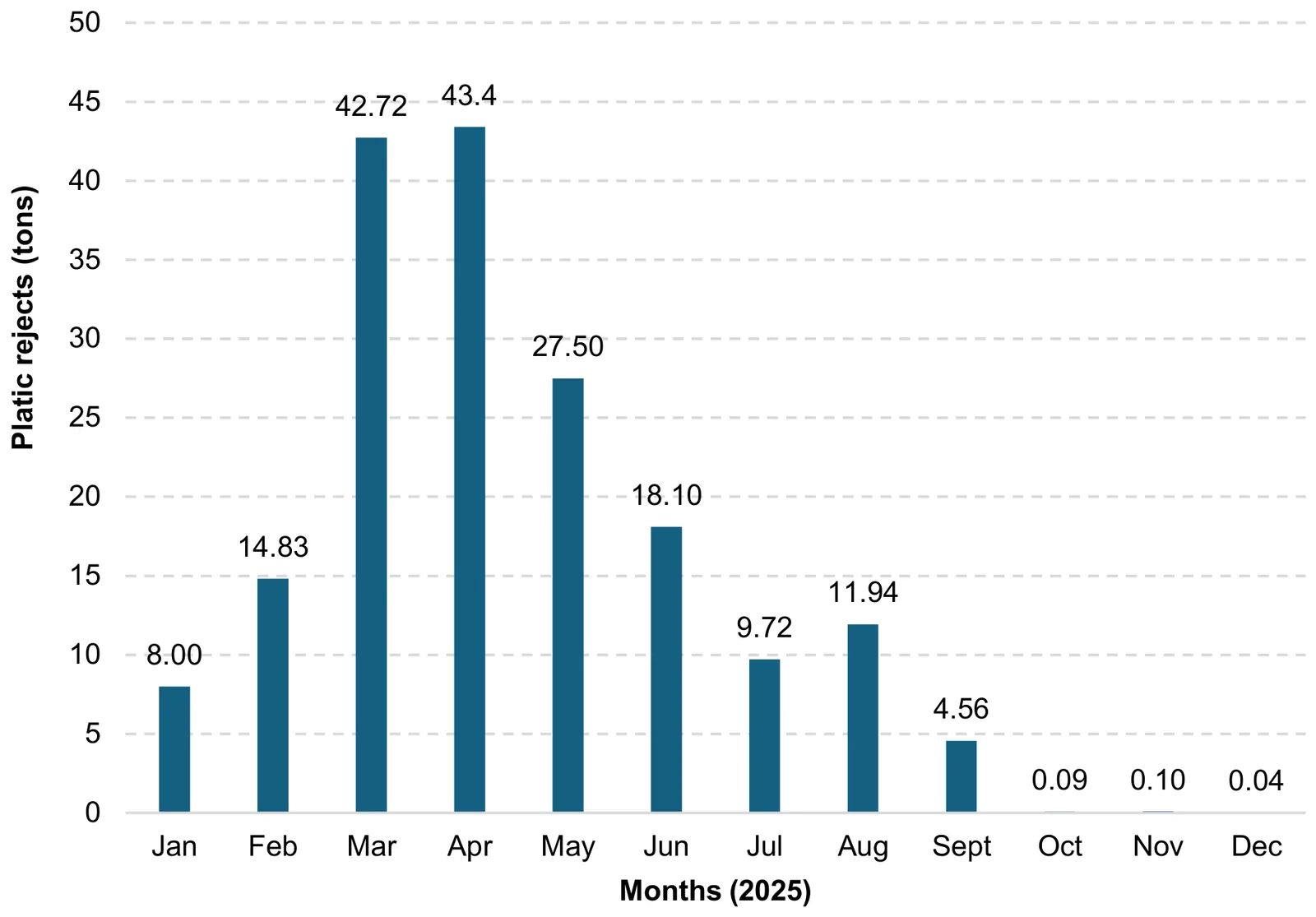
Plastic rejects (tons) at the Libegreen Innovation MRF in 2025.

**Figure 6:**
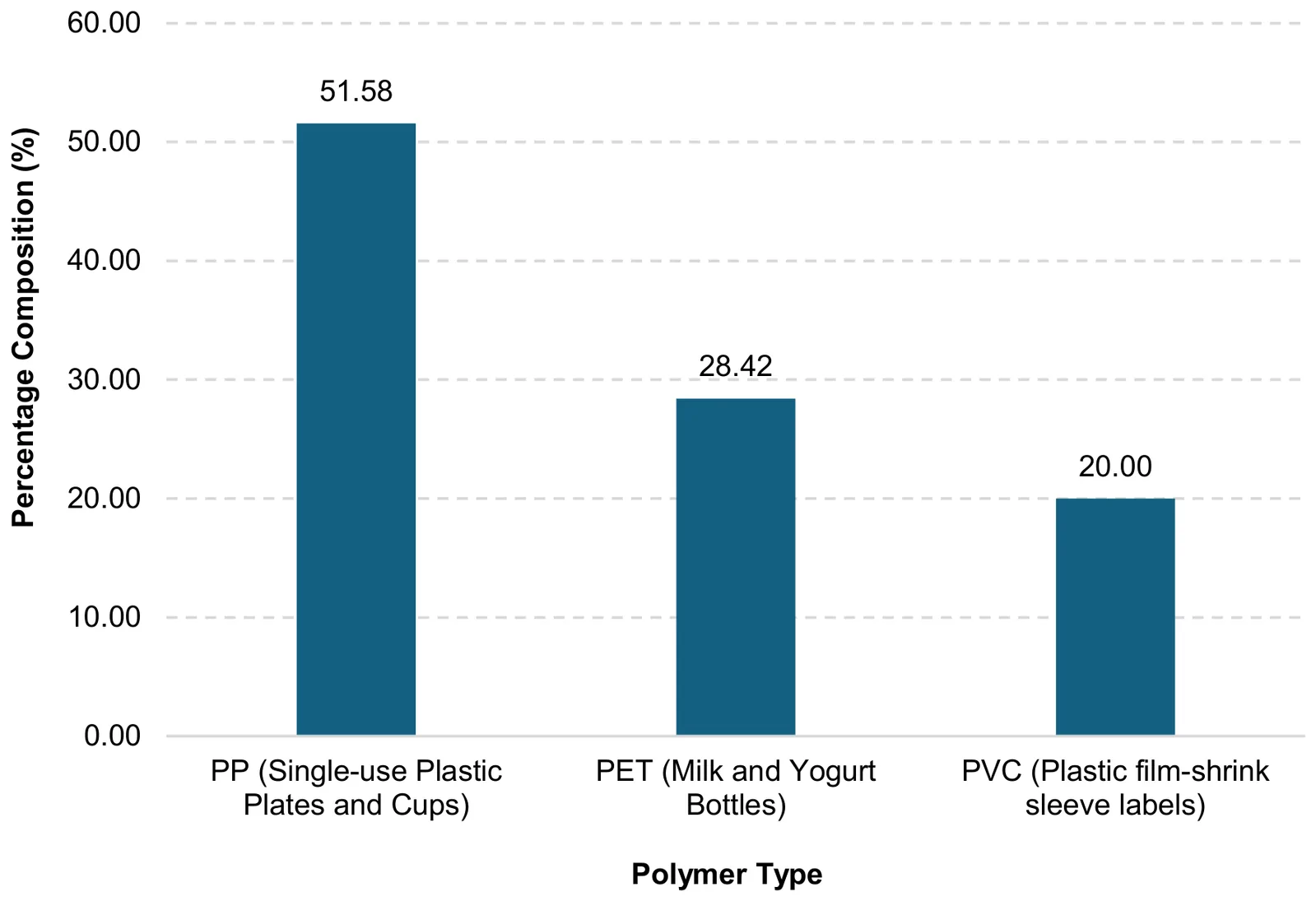
Polymer Composition of Plastic Rejects at Libegreen Innovation MRF.

Of the 4010 tons received, 95.5% (3829 tons) were sold to recyclers, whereas 4.5% (181 tons) were rejected. The facility generated an average plastic reject volume of 15.08 ± 15.39 tons per month, indicating a substantial monthly variation in rejection rates. The highest rejection volumes occurred in March (42.72 tons) and April (43.40 tons), as shown in Figure 5, which together accounted for approximately 47.6% of the total annual rejection. This rejection represented a substantial economic loss of approximately 90.5–163 million TZS ($34,774–62,632 USD) based on procurement costs of 300–900 TZS depending on the type of material. The reject stream was dominated by PP (51.58%), consisting mainly of single-use items such as disposable plates and cups. PET was followed at 28.42%; primarily contaminated milk and yogurt bottles were observed. PVC constituted the remaining 20%, mostly in the form of shrink-sleeve labels from plastic bottles (Figure 6).

### 3.2. Physical Characterization

#### 3.2.1. Proximate Analysis of Rejected Plastics

The proximate analysis of the plastic rejects in Table 1 reveals distinct variations in the thermal conversion characteristics among the polymer fractions. Polypropylene (PP) exhibited the highest volatile matter content (96.08%) and the lowest moisture (0.34%) and ash content (1.67%), indicating its high suitability for thermochemical conversion owing to its high hydrocarbon availability and minimal inorganic residue. Polyethylene terephthalate (PET) also exhibited a high volatile fraction (91.42%), with relatively low moisture (0.64%) and ash content (2.51%), suggesting favorable conversion potential, although its fixed carbon content (5.43%) was higher than that of PP. In contrast, polyvinyl chloride (PVC) demonstrated significantly different characteristics, with the highest moisture (7.12%), ash (9.01%), and fixed carbon (6.43%) contents and the lowest volatile matter (77.44%). The elevated ash fraction in PVC indicates greater inorganic additive loading and potential challenges during thermal processing, including increased residue formation and operational limitations. The mixed plastic fraction displayed intermediate characteristics, with 92.66% volatile matter, 3.68% ash content, 0.45% moisture, and 3.21% fixed carbon, reflecting the combined influence of the individual polymer constituents.

#### 3.2.2. Calorific Value Analysis of Rejected Plastics

The energy content of the plastic rejected from the material recovery facility was evaluated based on the bomb calorific value, higher heating value (HHV), and lower heating value (LHV), as presented in Table 2. The calorific values varied among the different fractions. Statistical evaluation showed that the bomb calorific value ranged from 20.9765 to 28.8515 MJ/kg, with an average value of 25.44 ± 3.33 MJ/kg, indicating moderate variability among the rejected plastics. Similarly, the HHV on a dry basis ranged between 20.9555 and 28.8227 MJ/kg, with a mean value of 25.42 ± 3.33 MJ/kg, whereas the LHV averaged 24.21 ± 3.34 MJ/kg. Polypropylene (PP) exhibited the highest energy content, with an HHV (dry basis) of 28.8227 MJ/kg and an LHV of 27.5358 MJ/kg, representing approximately 13.7% higher energy potential than the average LHV of the analyzed rejected plastics. The mixed plastic fraction also demonstrated energy recovery potential, with HHV and LHV values of 26.7063 MJ/kg and 25.6156 MJ/kg, respectively. Despite being an unsorted heterogeneous fraction, mixed plastics exhibited an LHV approximately 5.8% higher than the overall mean (24.21 MJ/kg), suggesting that rejected mixed plastic streams retain considerable fuel value. PET exhibited moderate energy characteristics, with an LHV of 23.9879 MJ/kg, which was close to the average value. In contrast, PVC exhibited the lowest energy content, with an HHV of 20.9555 MJ/kg and LHV of 19.7029 MJ/kg, approximately 18.6% lower than the mean LHV.

#### 3.2.3. Ultimate Analysis of Rejected Plastics

Ultimate analysis was conducted to determine the carbon (C), hydrogen (H), sulfur (S), and nitrogen (N) contents of the rejected plastic fractions. The results presented in Table 3 reveal substantial differences in elemental composition among the investigated materials. The rejected plastics from the material recovery facility were primarily composed of carbon and hydrogen. The carbon content varied from 47.44% to 73.7386%, with an average value of 61.71 ± 11.69%, while the hydrogen content ranged from 4.2680% to 11.2425%, averaging 8.39 ± 3.02% across the rejected plastic samples (Table 3). The inorganic elements and halogen contents, such as the high chlorine content found in PVC, were analyzed separately via X-ray fluorescence and are detailed in Table 4. Polypropylene (PP) exhibited the highest carbon concentration (73.7386%), corresponding with its superior calorific value and confirming its high energy recovery potential. PVC also recorded high carbon (68.5130%) and hydrogen (11.2425%) content. Mixed plastics contained moderate levels of carbon (57.1570%) and hydrogen. Nitrogen was not detected in any sample, whereas sulfur remained very low, with an average concentration of 0.19 ± 0.18%. This indicates a low risk of fuel-bound NO_X_ and SO_X_ emissions.

#### 3.2.4. X-Ray Fluorescence Analysis

The XRF results presented in Table 4 reveal compositional differentiation among the plastic reject fractions, reflecting substantial variations in inorganic additives, pigments, fillers, and contaminant loading. PET had the highest TiO_2_ concentration (47.7 wt%), contributing to the large variability observed for TiO_2_ across the polymer fractions (22.51 ± 18.14 wt%). In contrast, PVC exhibited the highest chlorine concentration (36.7 wt%), resulting in a considerable variation in the Cl content among the reject fractions (17.76 ± 12.82 wt%). The presence of Sb_2_O_3_ in PVC further suggests the incorporation of flame-retardant additives into the PVC. PP was dominated by mineral-associated oxides, particularly CaO (28.6 wt%). %), SiO_2_ (26.8 wt%), and Al_2_O_3_ (10.1 wt%), indicating a highly filled composite material. The moderate variability observed for CaO (18.48 ± 6.73 wt%) and SiO_2_ (18.75 ± 6.16 wt%) demonstrates differences in mineral filler loading among the polymer fractions, which may influence the ash generation and thermal processing behavior. The mixed plastic fraction displayed intermediate elemental characteristics, including TiO_2_ (23.1 wt%) and Cl (14.4 wt%), along with elevated Fe_2_O_3_ (7.17 wt%), originating from iron-based additives. This confirmed that the unsorted fraction represented a physical combination of the individual polymer streams.

#### 3.2.5. X-Ray Diffraction Analysis

**Figure 7:**
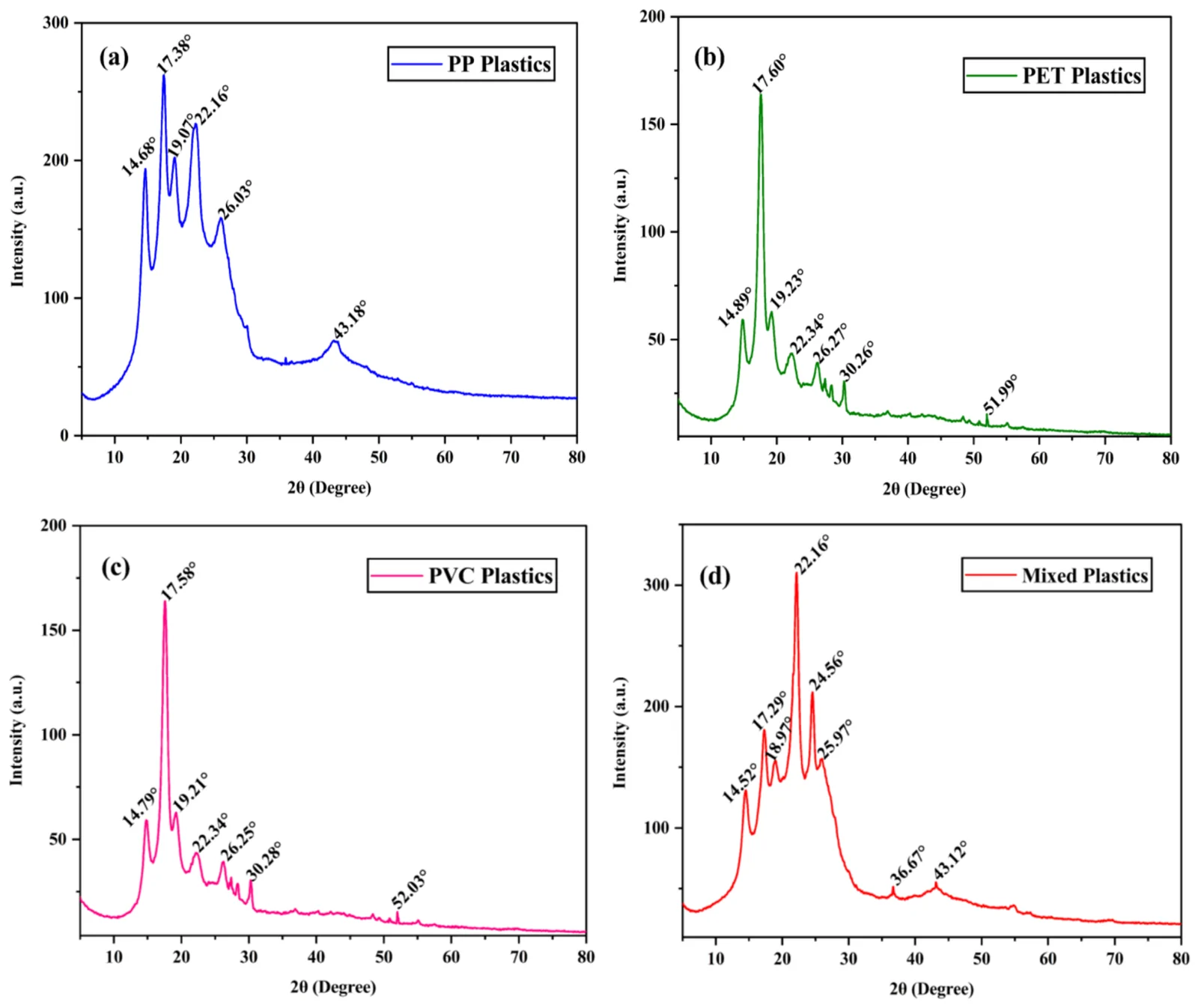
XRD patterns of the investigated plastic fractions. (**a**) XRD pattern of polypropylene (PP) plastics, showing the characteristic diffraction peaks of PP. (**b**) XRD pattern of polyethylene terephthalate (PET) plastics, showing the characteristic diffraction peaks of PET. (**c**) XRD pattern of polyvinyl chloride (PVC) plastics, showing the characteristic diffraction peaks of PVC. (**d**) XRD pattern of the mixed plastic fraction, showing the combined diffraction features of the constituent plastic polymers.

X-ray diffraction (XRD) analysis of the Libegreen Innovation material recovery facility (MRF) plastic rejects (Figure 7) revealed distinct differences in polymer crystallinity, confirming the heterogeneous physical structure of the waste streams. Among the individual polymer fractions, polyethylene terephthalate (PET) from post-consumer bottles exhibited the highest crystallinity, characterized by a dominant diffraction peak at 22.5°. Conversely, polypropylene (PP) displayed an intermediate crystalline order with a broader primary reflection peak between 14 and 25°, whereas polyvinyl chloride (PVC) exhibited the lowest crystallinity, peaking at 21.5°. Extensive chain branching along the PVC backbone drives structural suppression by hindering tight molecular packing. The unsorted mixed plastic rejects yielded a composite diffractogram between 21 and 23° that combined the individual diffraction patterns of its constituents rather than forming new crystalline phases. This profile demonstrates that the mixed reject stream behaves as a physical blend, where individual polymers retain their respective crystalline identities.

#### 3.2.6. Thermogravimetric Analysis

**Figure 8:**
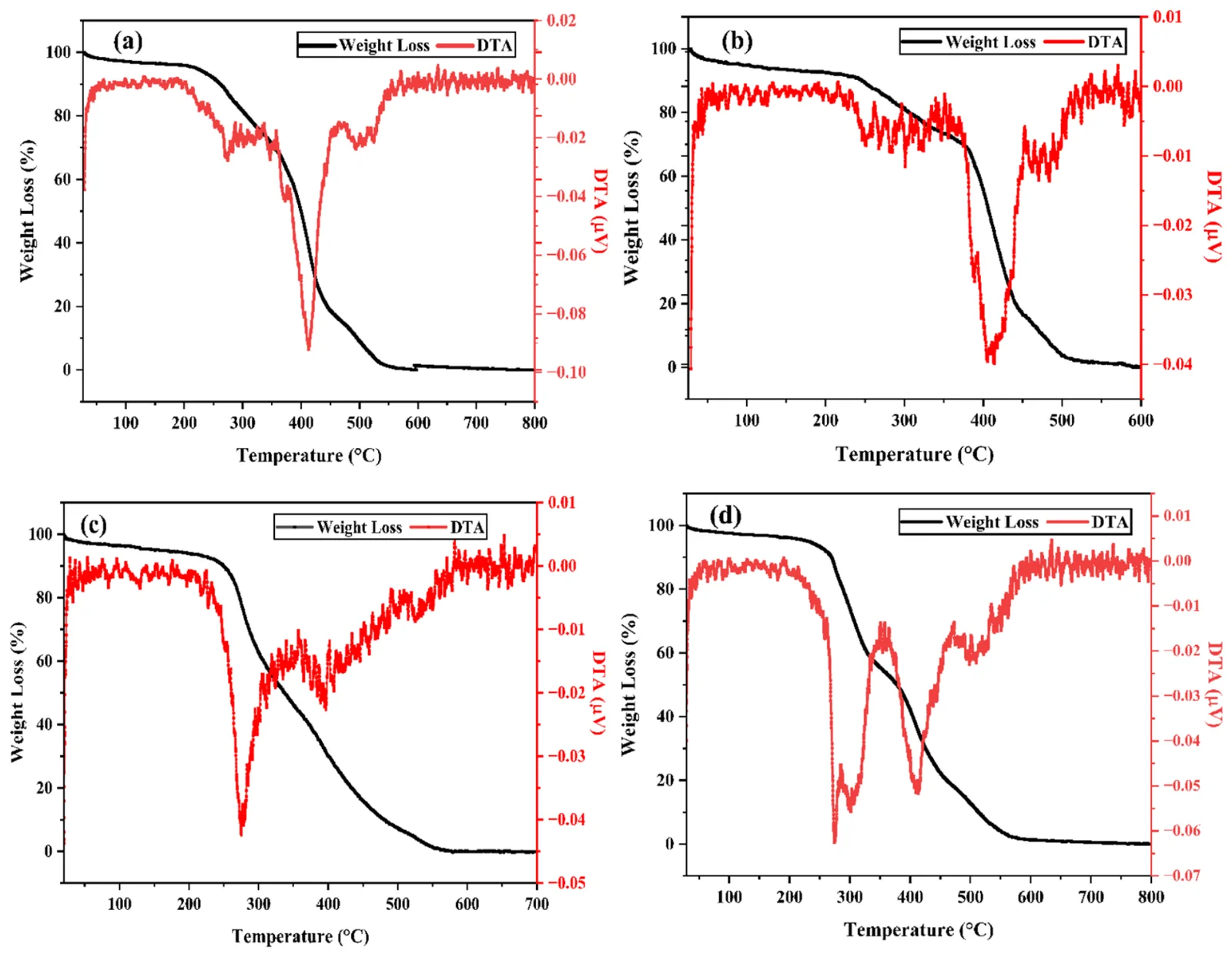
Thermogravimetric analysis (TGA) and derivative thermogravimetric analysis (DTA) profiles of the investigated plastic fractions. (**a**) TGA and DTA profiles of polypropylene (PP) plastics. (**b**) TGA and DTA profiles of polyethylene terephthalate (PET) plastics. (**c**) TGA and DTA profiles of polyvinyl chloride (PVC) plastics. (**d**) TGA and DTA profiles of the mixed plastic fraction.

TGA/DTA (Figure 8) revealed distinct thermal degradation behaviors among the PP, PET, PVC, and mixed plastic samples of the rejects. PP and PET exhibited single-stage degradation pathways. PP initiated significant weight loss at approximately 250 °C, whereas PET remained thermally stable until nearly 300 °C. Both polymers reached their maximum decomposition rates at approximately 410 °C, as confirmed by the pronounced endothermic DTA peaks. In contrast, PVC exhibited a characteristic multi-stage degradation pattern. An initial rapid weight loss occurred near 280 °C, corresponding to a major DTA peak, followed by a slower decomposition stage extending to approximately 550 °C. The mixed plastic sample exhibited a cumulative thermal degradation profile. A distinct degradation peak was observed near 280 °C, followed by a broader degradation region between 400 °C and 500 °C. The complete volatilization of the mixed sample was achieved at approximately 600 °C.

#### 3.2.7. Fourier Transform Infrared Spectroscopy (FT-IR)

**Figure 9:**
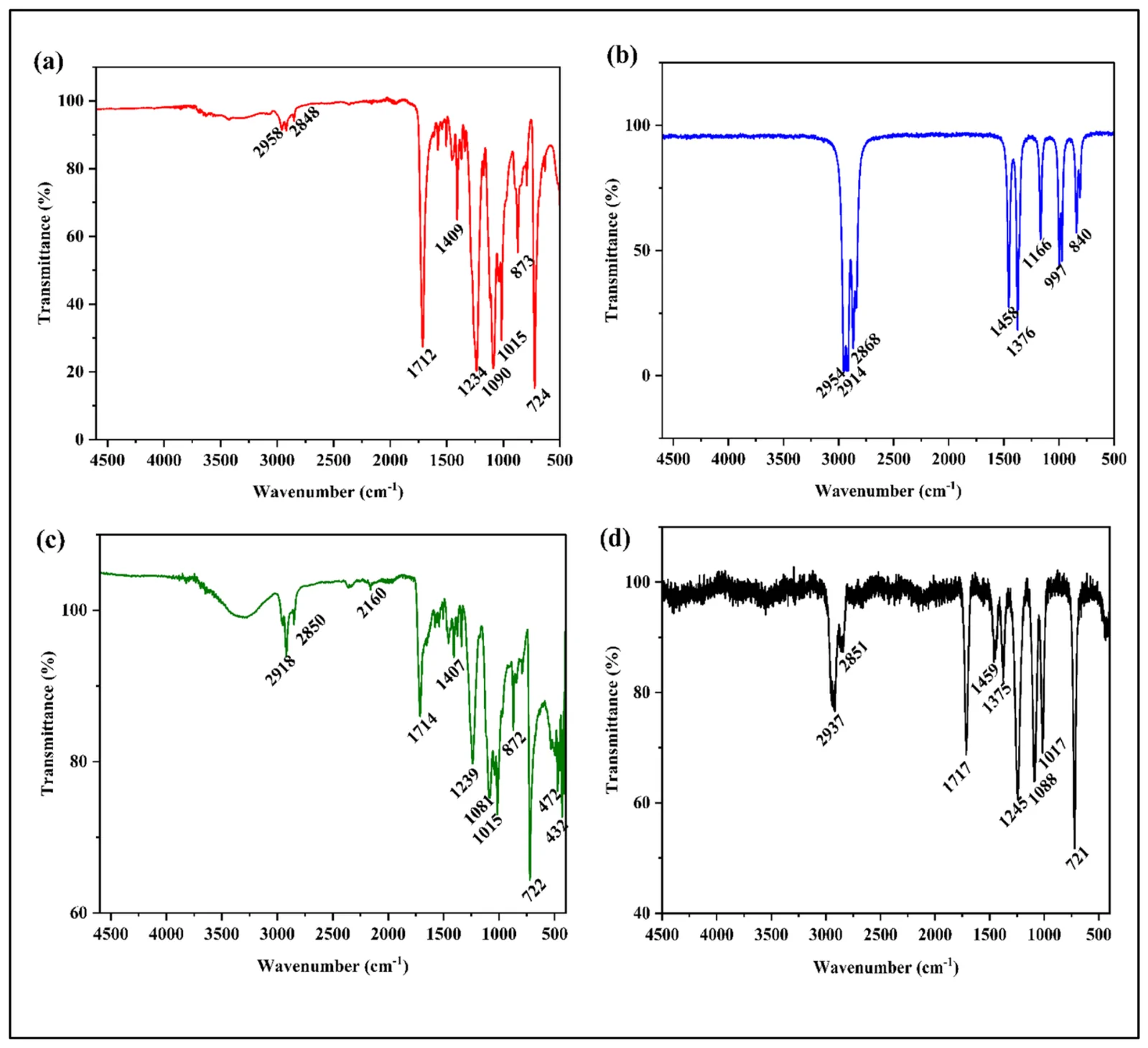
FTIR spectra of the investigated plastic fractions. (**a**) FTIR spectrum of polypropylene (PET). (**b**) FTIR spectrum of polyethylene terephthalate (PP). (**c**) FTIR spectrum of polyvinyl chloride (PVC). (**d**) FTIR spectrum of the mixed plastic fraction (MIXED).

Fourier-transform infrared (FT-IR) spectroscopic analysis was used to identify the chemical traits of the plastic rejects collected from Libe Green Innovation material recovery facility (Figure 9). The FTIR analysis of the PET reject material show a sharp ester carbonyl (C=O) band at 1713 cm^−1^, backed by strong C-O bonds at 1236 cm^−1^ and 1089 cm^−1^. Aromatic ring structures are also visible through bending points at 1015 cm^−1^ and 722 cm^−1^, alongside standard C-H links between 2850 and 2960 cm^−1^. These bands confirm an intact polymer backbone despite baseline scattering from an exceptionally high titanium dioxide concentration (47.7 wt%). The sampled PP reject plastics exhibited a prominent aliphatic C-H stretching modes at 2918 cm^−1^ and 2849 cm^−1^ alongside methyl (CH_3_) and methylene (CH_2_) bending configurations at 1456, 1409, 1353, and 1324 cm^−1^. Meanwhile, the PVC rejected materials displayed an overlapping aliphatic C-H and CH-Cl stretching at 2941, 2918, and 2850 cm^−1^, a methylene deformation band adjacent to chlorine at 1407 cm^−1^, and lower-range C-Cl carbon–halogen links at 722 cm^−1^ and 432 cm^−1^. These halogen features match the high elemental chlorine (36.7%) and flame-retardant antimony trioxide (0.343%) identified via XRF. In contrast, the unsorted mixed rejected plastics indicated an absorption band from each constituent polymer (PP/PET/PVC). The FTIR analysis indicates aliphatic C-H stretching peaks at 2937 cm^−1^ and 2851 cm^−1^, the PET ester carbonyl (C=O) stretches at 1717 cm^−1^, PP methyl (CH_3_) and methylene (CH_2_) deformation bands at 1459 cm^−1^ and 1375 cm^−1^, and ester C-O linkages at 1245 cm^−1^ and 1088 cm^−1^. Moreover, a sharp composite peak emerges at 721 cm^−1^, representing the overlapping out-of-plane aromatic C-H bend of PET and the C-Cl stretch of PVC. This indicates that the unsorted stream behaves as a physical blend. This is consistent with the elements identified by XRF (23.1 wt% TiO_2_, 14.4 wt% Cl, and 7.17 wt% Fe_2_O_3_), the intermediate proximate analysis values (92.66% volatile matter, 3.68% ash, and 26.71 MJ/kg HHV), and the multi-stage TGA curves indicating sequential PVC dehydrochlorination at 280 °C.

### 3.3. Multi-Criteria Decision Analysis (MCDA)

Physicochemical analysis indicated that the reject stream possesses diverse characteristics, including high levels of chlorine, significant amounts of inorganic fillers, and fluctuating calorific values. These features directly impact the technical viability, environmental effectiveness, economic recovery prospects, and operational appropriateness of chemical recycling methods. Consequently, a combined AHP-TOPSIS framework was employed to systematically integrate the experimentally obtained feedstock traits into the process of selecting suitable technologies.

#### 3.3.1. Analytical Hierarchy Process (AHP) and Criteria Weighting

The decision criteria were prioritized using a structured AHP by a panel of four experts. Their evaluations were based on the physical and chemical properties of 181 tons of annual plastic waste, focusing on the high chlorine content (36.7 wt%) in PVC and substantial inorganic fillers (over 65 wt%) in PP. The pairwise comparison matrix (Equation (1)) highlights the prioritization of Economic Feasibility (C2) and Environmental Impact (C3) to address an annual resource loss of $62,632 and associated emission risks. These criteria were chosen based on prior studies on waste management and resource recovery, which stressed the need to incorporate process efficiency, operational costs, environmental risks, and societal acceptance into technology selection frameworks [27]. The criteria weights were then calculated using the Row Geometric Mean (RGM) method, as presented in Table 5. The findings in Table 5 reveal that economic feasibility (C2) was the leading factor (48%), followed by environmental impact (C3) at 28%, technical performance (C1) at 17%), and social feasibility (C4) at 7%. Additionally, to verify these assessments, a consistency analysis was conducted, resulting in a principal eigenvalue (λ_max_) of 4.048 (Equation (2)). The Consistency Ratio (CR) was determined to be 0.0179 (Equation (3)), which is well below the 0.1 threshold. This low CR value, indicating less than 2% inconsistency, confirms the mathematical soundness and logical consistency of the panel’s prioritization, showing minimal deviation from perfect transitivity in the pairwise comparison judgments [28].

(1) $$A=\left[\begin{array}{cccc} 1 & a_{12} & \ldots & a_{1n}\\ a_{21} & 1 & \ldots & a_{2n}\\ \vdots & \vdots & \ddots & \vdots \\ a_{n1} & a_{n2} & \ldots & 1 \end{array}\right]=\left[\begin{array}{cccc} 1 & 1/3 & 1/2 & 3\\ 3 & 1 & 2 & 6\\ 2 & 1/2 & 1 & 4\\ 1/3 & 1/6 & 1/4 & 1 \end{array}\right]$$

(2) $${\lambda \;}_{\max}=\frac{\sum_{i=1}^{n}{\lambda \;}_{i}}{n}=4.048$$

(3) $$\mathrm{CI}=\frac{{\lambda \;}_{\max}-n}{n-1}=\frac{4.048-4}{4-1}=0.016\to \;\mathrm{CR}=\frac{\mathrm{CI}}{\mathrm{RI}}=\frac{0.016}{0.90}=0.0179$$

#### 3.3.2. Technology Ranking via TOPSIS

The evaluation of three technological options, namely pyrolysis (A1), gasification (A2), and depolymerization (A3), was conducted by a targeted expert panel of four members, including three academic researchers and one industry professional from Libegreen Innovation. To maintain reproducibility, the decision scores in the Initial Decision Matrix, as shown in Table 6, were determined by directly correlating the technological capabilities with the empirical laboratory findings. Gasification (A2) received a technical performance score of 9 due to its demonstrated resilience against high inorganic filler levels, identified via XRF, which surpassed 65 wt% in PP and reached 47.7 wt% TiO_2_ in PET. Pyrolysis (A1) was awarded a score of 9 for economic feasibility, given that the 28.82 MJ/kg High Heating Value (HHV) of polypropylene offers the greatest potential to counterbalance the facility’s $62,632 annual resource deficit. However, pyrolysis was environmentally penalized with a score of 3 because of the presence of 36.7 wt% chlorine in the PVC rejects and TGA-confirmed dehydrochlorination at 280 °C. Any scoring disagreements were resolved through a consensus-building workshop, in which members relied on primary physical and chemical laboratory evidence. Because the criteria were measured in various units, from technical ash content (9.01% in PVC) to economic heating values, the raw scores were standardized using the vector normalization formula.

The TOPSIS analysis results in Table 7 highlight gasification (A2) as the most suitable chemical recycling technology for the Libegreen Innovation MRF, achieving a superior closeness coefficient (R_i_) of 0.718. This choice is primarily influenced by the AHP-derived criteria weights, where Economic Feasibility (C2, 47.2%) and Environmental Impact (C3, 28.5%) were identified as key factors in mitigating the US$62,632 annual resource loss and managing the 36.7 wt% Cl content in the plastic reject stream. These criteria were treated as benefit criteria, with gasification securing its top position by maintaining the shortest geometric distance to the Positive Ideal Solution (d_i_^+^ = 0.07). This highlights the unique ability of the technology to handle rejects characterized by inorganic fillers exceeding 65 wt% and 47.7 wt% TiO_2_ in PET. In contrast, pyrolysis (A1) and depolymerization (A3) achieved lower R_i_ values of 0.517 and 0.196, respectively, as they showed greater geometric proximity to the Negative Ideal Solution (A^−^), indicating a lower suitability for the facility’s diverse 181-ton annual reject stream.

#### 3.3.3. Sensitivity Analysis

To verify the model robustness, a sensitivity analysis was conducted to evaluate the impact of varying the emphasis placed on the US$62,632 annual resource loss relative to technical and environmental criteria. Decreasing the initial economic feasibility weight (w2) from 47.2% and redistributing the difference to technical feasibility (w1) and environmental impact (w3) yielded a revised criteria weight vector (w1, w2, w3, w4) of (0.234, 0.300, 0.393, 0.073). As detailed in Table 8, gasification (A2) secured its top ranking as its closeness coefficient increased from 0.718 to 0.910 (+0.192). This performance trajectory under shifting weight priorities confirms the model stability and ensures high confidence in the evaluation. Furthermore, the enhanced performance of gasification under stricter technical and environmental constraints underscores its operational resilience to complex feedstocks with elevated chlorine concentrations (36.7 wt% Cl) and substantial inorganic filler fractions. Conversely, pyrolysis (A1) and depolymerization (A3) consistently held their respective second and third positions, confirming that the established technology hierarchy remains valid for specific operational streams at the Libegreen Innovation MRF.

## 4. Discussion

The Libegreen Innovation MRF demonstrated an exceptionally low rejection rate of 4.5% (181 tons) relative to its 4010-ton annual inflow. Although this rejected fraction represents lost procurement expenditures and threatens environmental pollution if released into the ecosystem, this operational efficiency significantly surpasses the benchmarks in high-income regions. For comparison, the MRFs in Spain and Italy reported rejection rates of 20–30% and 34–50%, respectively [29,30]. Similarly, these findings exceed the recovery efficiencies documented in Brazil, where 29% losses occur in HDPE chains [31]. This disparity suggests that labor-intensive manual sorting in Tanzania is highly effective at capturing primary recyclables, driven by the economic necessity for waste pickers to maximize recovery. Remarkably, 62.7% of the material inflow occurred during the rainy season (March–May), challenging the assumption that weather is the primary hindrance to recovery. This aligns with the findings of [32] and [33] in South Africa, suggesting that seasonal climatic variations do not reliably predict recovery rates. Instead, the sharp decline in deliveries between October and December highlights the impact of sociopolitical factors, specifically disruptions following election periods. This pattern suggests that plastic waste management in Tanzania depends more on administrative stability and implementation capacity than on environmental conditions. This aligns with previous studies that identified governance, institutional capacity, and administrative stability as critical drivers of waste management performance [34,35,36,37,38]. However, the extent to which these governance-related factors influence fluctuations in plastic waste collection and delivery remains unclear. These fluctuations in waste delivery disrupt the continuity of material recovery, sorting-section worker livelihoods, and the volume of residual plastics requiring disposal. Therefore, improving MRF performance requires both technical recovery capacity and stable institutional coordination.

The high volatile matter content (77.44–96.08%) across all reject streams confirms their high potential for thermochemical conversion. Consistently, empirical characterizations by [6,39] link high volatile matter and low ash fractions to elevated thermal decomposition rates, whereas [40] designated polyolefin-based plastics as preferred feedstocks for thermochemical recovery. Specifically, the high volatile matter (96.08%) and calorific value (28.82 MJ/kg) of polypropylene yield a large carbon fraction available for conversion into combustible gases or liquid fuels. These properties enhance the energy recovery efficiency and economic feasibility, driving pyrolysis to the highest economic score in the MCDA, notwithstanding its lower environmental performance. Consistent with these findings, the high carbon and hydrogen content aligns with [29,39], while the minimal nitrogen and sulfur levels (0.19%) match those of [41], confirming low risks of fuel-bound NO_X_ and SO_X_ emissions. Consequently, these rejects represent a valuable chemical and energy resource, rather than a mere disposal burden. Recognizing this allows for a strategic shift to reduce the societal impacts of landfilling and open burning by routing unrecyclable residues into optimized recovery pathways.

The thermal degradation patterns further delineate the challenges of mixed rejects. The single-stage pathways observed for PP and PET are consistent with thermogravimetric and kinetic characterization profiles reported by [42,23]. The onset of PVC degradation at approximately 280 °C corresponds to dehydrochlorination, which releases hydrogen chloride (HCl) before complete polymer decomposition. This process accelerates reactor corrosion, increases gas cleaning requirements, and lowers the environmental suitability of chlorine-rich feedstocks for conventional pyrolysis. Furthermore, FTIR spectroscopic band identification such as PET ester C=O at 1713 cm^−1^ and PVC C-Cl stretching at 722 cm^−1^ and XRF elemental signatures confirmed that inorganic fillers (CaO, SiO_2_) and pigments (TiO_2_ up to 47.7 wt%) are heavily incorporated into the reject stream. Such high inorganic additive loadings and mixed polymer blending are widely documented to reduce volatilization rates, alter reaction kinetics, and increase slagging during thermochemical conversion [43,44]. Specifically, elevated chlorine concentrations, high inorganic filler content, and heterogeneous polymer compositions reduce the viability of technologies that require uniform feedstocks. To mitigate these operational issues, chlorine management can be achieved by integrating feedstock pretreatments, such as mechanical sorting or staged thermal dehydrochlorination at 250–350 °C, with downstream gas scrubbing using alkaline solutions like NaOH, Na_2_CO_3_, or Ca (OH)_2_ to neutralize volatile HCl [45,46]. The AHP-TOPSIS assessment identified gasification (Ri = 0.718) as the optimal technology, which is consistent with the findings of [47,48], who advocated the use of multi-criteria frameworks to integrate technical indicators with expert judgment. Because gasification tolerates heterogeneous waste streams, it maintains superior technical and environmental performance, resulting in the maximum overall TOPSIS closeness coefficient. Importantly, the evaluation framework embedded experimentally determined characteristics of Tanzanian MRF rejects instead of assuming geographical equivalence in waste stream properties. Incorporating these measured criteria, including composition, calorific value, thermal behavior, and contaminant-related constraints, ensured that the technology ranking reflected empirical realities rather than subjective preferences. Consequently, relying exclusively on international performance data risks overlooking the distinctive feedstock characteristics and infrastructure constraints that dictate practical feasibility in Tanzania.

## 5. Conclusions

This study presents an integrated characterization and technology assessment of plastic rejects generated by an operational Material Recovery Facility in Dar es Salaam, Tanzania. The findings demonstrate that although manual sorting achieved high recovery efficiency, diverting 95.5% of incoming plastic streams to recyclers, the remaining 4.5% reject fraction represents both an environmental burden and an economic loss estimated at USD 34,774–62,632 annually to one plastic-focused material recovery facility. The rejected fraction was primarily composed of PP, PET, and PVC, highlighting the limitations of mechanical recycling in handling contaminated, additive-rich, and low-value plastic streams. Moreover, physicochemical characterization revealed that the rejects were not pure polymer fractions but formulation-driven composite materials containing significant amounts of inorganic fillers, pigments, and additives. PP demonstrated the highest thermochemical potential due to its high volatile content and calorific value, whereas PVC introduced operational constraints associated with chlorine release during thermal degradation. Thermal analysis further confirmed that mixed plastic rejects behave as physical blends with sequential degradation patterns, requiring robust conversion technologies. Based on the evaluated technical, economic, environmental, and social criteria, gasification achieved the highest TOPSIS closeness coefficient and was therefore ranked first among the considered technologies. These findings demonstrate that chemical recycling can provide a viable pathway for recovering value from currently discarded plastics while supporting the development of a circular economy. This study addresses a critical data gap in African plastic waste management and provides a decision-support framework for implementing sustainable waste-to-resource technologies in rapidly urbanizing regions.

## Figures and Tables

**Table 1:** Proximate analysis of rejected plastics.

Polymer Type	Moisture	Ash Content A (%)		Volatile Matter V (%)			Fixed Carbon
	M_ad_ (%)	A_ad_	A_d_	V_ad_	V_d_	V_daf_	FC_ad_ (%)
PP	0.34	1.67	1.68	96.08	96.41	98.05	1.91
PET	0.64	2.51	2.52	91.42	92.01	94.39	5.43
PVC	7.12	9.01	9.70	77.44	83.38	92.33	6.43
Mixed Plastics	0.45	3.68	3.70	92.66	93.08	96.65	3.21

Note: M_ad_ means Moisture content on air-dried basis, A_ad_ means Ash content on air-dried basis, A_d_ means Ash content on dry basis, V_ad_ means Volatile matter on air-dried basis, V_d_ means Volatile matter on dry basis, V_daf_ means Volatile matter on dry ash-free basis, and FC_ad_ means Fixed carbon on air-dried basis.

**Table 2:** Calorific Value of rejected plastics.

Polymer Type	Bomb Calorific Value Q_b,ad_ (MJ/g)	Higher Heating Value (HHV) Q_gr,v,ad_ (MJ/g)	Higher Heating Value (HHV) Qgr_,v,d_ (MJ/g)	Lower Heating Value (LHV) Qnet_,v_, M_ad_ (MJ/g)
PP	28.8515	28.9646	28.8227	27.5358
PET	25.2185	25.2639	25.1933	23.9879
PVC	20.9765	22.0713	20.9555	19.7029
Mixed Plastics	26.7330	26.8484	26.7063	25.6156

Note: ad-air-dried, basis d-dry basis. The subscripts b, gr, v, and net represent bomb, gross, constant volume, and net calorific value respectively.

**Table 3:** Ultimate Analysis of rejected plastics.

Polymer Type	N (%)	C (%)	H (%)	S (%)
PP	0	73.7386	10.3510	0.2066
PET	0	47.4400	7.88210	0.0000
PVC	0	68.5130	11.2425	0.0000
Mixed Plastics	0	57.1570	4.26880	0.3694

**Table 4:** XRF oxide composition (wt%) of rejected plastics.

Elements (%)	PP	PET	PVC	Mixed Plastics
TiO_2_	5.84	47.70	13.40	23.10
SiO_2_	26.8	15.30	12.70	20.20
CaO	28.6	13.10	15.30	16.90
Cl	11.9	8.050	36.70	14.40
Fe_2_O_3_	5.93	4.110	2.350	7.170
MgO	5.55	2.460	2.260	2.760
P_2_O_5_	1.50	1.110	-	0.917
Al_2_O_3_	10.1	-	7.410	6.750
Na_2_O	-	-	2.700	3.600
SO_3_	1.40	-	3.570	2.000
K_2_O	-	0.633	1.370	1.170
Cr_2_O_3_	0.00	0.000	-	0.629
CuO	-	-	-	0.166
Sb_2_O_3_	0.00	0.000	0.343	0.000
CeO_2_	-	-	0.317	-
Sc_2_O_3_	-	-	0.155	-

**Table 5:** Derivation of AHP criteria weights using the Row Geometric Mean (RGM) method.

Decision Criteria	ri=∏j=1naij1n	$w_{i}=\frac{r_{i}}{\sum_{i=1}^{n}r_{i}}$	Weight (%)
C1: Technical Performance	0.84	0.167	17
C2: Economic Feasibility	2.45	0.486	48
C3: Environmental Impact	1.41	0.280	28
C4: Social Feasibility	0.34	0.067	07
SUM	$\sum r_{i}$ = 5.04	1.0	100

Note: w_i_ represents weight normalization, C1 represent technical performance, C2 represent economic feasibility, C3 represents environmental impact, and C4 represents social feasibility.

**Table 6:** Initial and normalized decision matrices for the evaluated chemical-recycling technologies.

Technological Alternative (Chemical Recycling of Plastics Rejects)	Initial Decision Matrix (X)				Normalized Decision Matrix (R) = $\frac{x_{\mathrm{ij}}}{\sqrt{\sum_{i=1}^{m}x_{\mathrm{ij}}^{2}}}$			
	C1	C2	C3	C4	C1	C2	C3	C4
A1: Pyrolysis	6	9	3	7	0.545	0.695	0.303	0.573
A2: Gasification	9	8	8	8	0.818	0.617	0.808	0.655
A3: Depolymerization	2	4	5	6	0.182	0.309	0.505	0.491

**Table 7:** Weighted Normalized Decision Matrix, Criteria Weights (*w_j_*), Ideal Solutions, and Closeness coefficient.

Technological Alternative/Parameter	Decision Criterion				*d*_i_^+^ = $\sqrt{\sum {\left(v_{\mathrm{ij}}-A_{j}^{+}\right)}^{2}}$	*d*_i_^−^ = $\sqrt{\sum {\left(v_{\mathrm{ij}}-A_{j}^{+}\right)}^{2}}$	*CC_i_* = $\frac{d_{i}^{-}}{d_{i}^{-}+d_{i}^{+}}$	Rank
	C1	C2	C3	C4				
A1: Pyrolysis	0.093	0.328	0.086	0.042	0.15	0.16	0.517	2
A2: Gasification	0.146	0.291	0.230	0.049	0.07	0.18	0.718	1
A3: Depolymerization	0.032	0.164	0.144	0.035	0.05	0.21	0.196	3
Criteria Weights (*w_j_*)	0.170	0.472	0.285	0.073	-	-		
Positive Ideal (*A*^+^)	0.146	0.328	0.230	0.049	-	-		
Negative Ideal (*A*^−^)	0.032	0.164	0.086	0.035	-	-		

Note: A^+^ and A^−^ represent the maximum and minimum values derived from the weighted normalized matrix, di^+^ and di^−^ represent the Euclidean distances to the positive and negative ideal solutions, respectively; CC_i_ is the TOPSIS closeness coefficient used to rank the alternative, C1 represents technical performance, C2 represents economic feasibility, C3 represents environmental impact, and C4 represents social feasibility.

**Table 8:** Sensitivity analysis of TOPSIS ranking under revised criteria weights.

Technological Alternative (Chemical Recycling of Plastics Rejects)	Original Closeness Coefficient (*R_i_*)	Revised Closeness Coefficient $\left(R_{i}^{\prime }\right)$	Change $\left(R_{i}^{\prime }-R_{i}\right)$	Rank
A1: Pyrolysis	0.517	0.365	−0.152	2
A2: Gasification	0.718	0.910	+0.192	1
A3: Depolymerization	0.196	0.262	+0.066	3

## Data Availability

The data supporting the findings of this study are available from the corresponding author upon reasonable request.

## Abbreviations

The following abbreviations are used in this manuscript:

MRF	Material Recovery Facility
PP	Polypropylene
PET	Polyethylene terephthalate
PVC	Polyvinyl chloride
HDPE	High-density polyethylene
LDPE	Low-density polyethylene
PE	Polyethylene
FTIR	Fourier Transform Infrared Spectroscopy
XRF	X-ray Fluorescence
XRD	X-ray Diffraction
TGA	Thermogravimetric Analysis
DTA	Differential Thermal Analysis
MCDA	Multi-Criteria Decision Analysis
AHP	Analytical Hierarchy Process
TOPSIS	Technique for Order Preference by Similarity to Ideal Solution
RGM	Row Geometric Mean
HHV	Higher Heating Value
LHV	Lower Heating Value
FC	Fixed Carbon
Cl	Chlorine
CR	Consistency Ratio
USD	United States Dollar
TZS	Tanzanian Shilling
